# A novel *FGF23* mutation in hyperphosphatemic familial tumoral calcinosis and its deleterious effect on protein O-glycosylation

**DOI:** 10.3389/fendo.2022.1008800

**Published:** 2022-09-23

**Authors:** Qingyao Zuo, Weili Yang, Baoyue Liu, Dong Yan, Zhixin Wang, Hong Wang, Wei Deng, Xi Cao, Jinkui Yang

**Affiliations:** ^1^ Department of Endocrinology, Beijing Jishuitan Hospital, Beijing, China; ^2^ Beijing Diabetes Institute, Department of Endocrinology, Beijing Tongren Hospital, Capital Medical University, Beijing, China; ^3^ Department of Pathology, Beijing Jishuitan Hospital, Beijing, China; ^4^ Department of Radiology, Beijing Jishuitan Hospital, Beijing, China

**Keywords:** tumoral calcinosis, mutation, hyperphosphatemia, fibroblast growth factor 23, glycosylation

## Abstract

**Background:**

Hyperphosphatemic familial tumoral calcinosis (HFTC) is a rare disease characterized by hyperphosphatemia and ectopic calcification, predominantly at periarticular locations. This study was performed to characterize the clinical profile of tumoral calcinosis and to identify gene mutations associated with HFTC and elucidated its pathogenic role.

**Methods:**

The three subjects (two male and one female) were aged 30, 25 and 15 years, respectively. The clinical features, histopathological findings, and outcomes of three subjects with HFTC were retrospectively reviewed. The three subjects were analyzed for *FGF23*, *GALNT3* and *KL* mutations. Function of mutant gene was analyzed by western blotting and wheat germ agglutinin affinity chromatography.

**Results:**

All subjects had hyperphosphatemia and elevated calcium-phosphorus product. Calcinosis positions included the left shoulder, left index finger, and right hip. Bone and joint damage were present in two cases and multiple foci influenced body growth in one case. The histopathological features were firm, rubbery masses comprising multiple nodules of calcified material bordered by the proliferation of mononuclear or multinuclear macrophages, osteoclastic-like giant cells, fibroblasts, and chronic inflammatory cells. The novel mutation c.484A>G (p.N162D) in exon 3 of *FGF23* was identified in one subject and his family members. Measurement of circulating FGF23 in the subject confirmed low intact FGF23 and increased C-terminal fragment. *In vitro* experiments showed that the mutant FGF23 proteins had defective O-glycosylation and impaired protein proteolysis protection.

**Conclusion:**

We identified a novel *FGF23* missense mutation, and confirmed its damaging role in FGF23 protein O-glycosylation. Our findings expand the current spectrum of *FGF23* variations that influence phosphorus metabolism.

## Introduction

Tumoral calcinosis is a rare disorder characterized by ectopic calcification, predominantly at periarticular locations (1, 2), that can severely affect daily living and quality of life. Primary tumoral calcinosis is classified as hyperphosphatemic or normophosphatemic (3), while secondary tumoral calcinosis is often associated with chronic renal failure, hyperparathyroidism, and autoimmune diseases. Hyperphosphatemic familial tumoral calcinosis (HFTC) (online Mendelian inheritance in man (OMIM) 211900) is an autosomal recessive disease caused by mutations in the genes encoding the following proteins: fibroblast growth factor 23 (*FGF23*, 12p13.3), UDP-GalNAc: polypeptide N-acetylgalactosaminyltransferase-T3 (*GALNT3*), and Klotho (*KL*) (1, 4–6). Hyperphosphatemia and characteristic imaging features are the diagnostic hallmarks of HFTC (7). However, many other lesions have similar radiographic appearances, making diagnosis difficult by imaging alone. Reports of tumoral calcinosis in East Asia are very limited (8–10), and the exact prevalence is unknown. Here, we describe three Chinese patients with HFTC and identify a novel mutation in *FGF23*, and elucidated the pathogenic role of glycosylation deficiency in this disease.

## Materials and methods

### Subjects

Between January 2010 and March 2017, three patients with HFTC were admitted to Beijing Jishuitan Hospital. Renal failure, hyperparathyroidism, and autoimmune diseases were excluded. A retrospective review of these patients was performed and their biochemical data were obtained from medical records. All three cases were proven by histopathology and independently reconfirmed by two senior pathologists.

### Biochemical parameters

Relevant biochemical variables, including serum phosphorus, calcium, alkaline phosphatase, 25-hydroxyvitamin D [25-(OH)D], and parathyroid hormone, were extracted and recorded. A spot urine sample had been collected from the proband to assess the tubular reabsorption of phosphorus. The tubular maximum reabsorption rate of phosphate to glomerular filtration rate (TmP/GFR) was calculated by the Walton-Bijvoet nomogram (11). The plasma concentrations of intact FGF23 and the FGF23 C-terminal fragment were assessed using enzyme-linked immunosorbent assay kits (Immunotopics, San Clemente, CA, USA). For comparison, 10 healthy adults were selected as the control group.

### Genetic analysis

Genomic DNA was extracted from peripheral blood samples of the three patients and family members of Patient 1 using a HiPure Tissue & Blood DNA Kit (Magen, Guangzhou, China) according to the manufacturer’s instructions. All three *FGF23* exons, 10 *GALNT3* coding exons, and five *KL* exons including intron-exon boundary regions were PCR-amplified with specific sets of upstream and downstream primers (12, 13). Primer sequences are shown in **Table 1**. PCR amplification products were purified and sequenced using an automated sequencer (ABI 3730XL; Applied Biosystems, Foster City, CA, USA).

**Table 1:** Primers for the amplification of *FGF23*, *GALNT3*, and *KL*.

Primer name	Forward primers (3’-5’)	Reverse primers (3’-5’)
*FGF23* exon 1	AATCTCAGCACCAGCCACTC	GATGGACAACAAGGGTGCTC
*FGF23* exon 2	TTTCAGGAGGTGCTTGAAGG	TTGCAAATGGTGACCAACAC
*FGF23* exon 3-5'	AGGCTCAACGCCCTAAGAACT	ATGGGGGTGTTGAAGTGAATTAG
*FGF23* exon 3-3'	CTCTCCTCAGTATCACTTCCTGGT	TCGGAACGTCAAGGGACCT
*GALNT3* exon 2	CCATCGATCATTTCTGTTTATAGG	TCCTTAGCTCACCCCTCTCTC
*GALNT3* exon 3	CTCTGGGTGAGTGATTTGCTTG	CTGAGATGGCATACAGAGAGTAC
*GALNT3* exon 4	GCTCTGTGGTTTCATTAGCTTTC	CACAGAGCTGTTACCTGCTTGG
*GALNT3* exon 5	CAATAAATCTGAGGAAGAAAGAA	GCTATAAAGCAAACAGTGTGTAC
*GALNT3* exon 6	CAATGGGAGAGGACACGAAG	ACCAGCCGATTAGAACACAA
*GALNT3* exon 7	ATGGCAGGGGACAGAGACTA	ATGAATCGACGCAAAAGGAC
*GALNT3* exon 8/9	GGCTGTTGAATTGCCTCTTG	AGGCAACATCTCACTTGTGCT
*GALNT3* exon 10	GGCTATTGTATCGTCTATCAC	GATATATTCTCTTATCACATGGG
*GALNT3* exon 11	TCAGACATGGCTCACCTTAGAA	TTTAGCTGCTTTTGCATAATTTTC
*KL* exon 1	CAGGCAAAGAGAATGAACCT	CTCTCCTAATTCCACGCCTT
*KL* exon 2	TGCATTTCTCCTCACAACTAGA	ATTGCCAAAATGAATGTCTCCAT
*KL* exon 3	GAAACGCTCAGCTGCTCTTG	GCTTGGTGAGACTGCTGATT
*KL* exon 4-5'	GACGCTAATGTTTACTCTGC	TCAGCCAGTCCCTCATCACC
*KL* exon 4-3'	GGATAACGATGAATGAGCCG	GGATTTCTGGTCTTCTACTT
*KL* exon 5-1	AAAGTGATGTGTTGTGTGCAA	CGATCACCTATGCCCATTTCA
*KL* exon 5-2	GCTGTTAACCATTTGCACCTCTA	CAAGGCCCTCAACAAGATGC
*KL* exon 5-3	TGAGGTCCTGTCTAAACCCTG	AGCTCCAGTGTAATAGAGAGACT
*KL* exon 5-4	CACGCTGAAACATGCTAGTGA	CCACTGCTCCCATCACATCT

The obtained sequences were aligned against the human genome reference sequence hg38 (GRCh38; https://blast.ncbi.nlm.nih.gov/Blast.cgi). The possible functional significance of the potential candidate variant was assessed using Mutation Taster (http://www.mutationtaster.org/) and Polymorphism Phenotyping (PolyPhen-2; http://genetics.bwh.harvard.edu/pph2/). The impact of the variant on protein structure was further predicted by SWISS-MODEL (https://www.swissmodel.expasy.org). The variant was compared with the Human Gene Mutation Database (http://www.hgmd.cf.ac.uk) and ClinVar (https://www.ncbi.nlm.nih.gov/clinvar) to exclude previous reports.

### *In vitro* expression of mutant FGF23 and western blot analysis

c.484A>G mutation was introduced into the pcDNA3.1-FGF23 WT plasmid by using the fast mutagenesis system (TransGen Biotech, Beijing, China). A 6×His tag was cloned into the FGF23 C-terminal region. The related expression plasmid or empty vector was transiently transfected into hFOB1.19 osteoblast cells (National Experimental Cell Resource Sharing Platform, Beijing, China), and Lipofectamine 2000 transfection reagent (Invitrogen; Thermo Fisher Scientific, Inc) was used to perform the transfection. 24 hours after transfection, the medium was replaced with serum-free medium and cultured for another 24 hours. The cell lysate and medium (concentrated by 20-fold) were collected and analyzed for FGF23 protein expression by Western blot with rabbit anti-6X His tag antibody (Abcam, Cambridge, MA, ab9108, 1:1000).

### Western blot

After treatment, cells were collected and lysed with RIPA lysis buffer (Beyotime Biotechnology, China) containing 25 mmol/L protease inhibitor cocktail (Roche, Switzerland) and 100 mmol/L phenylmethylsulfonyl fluoride (Beyotime Biotechnology, China) on ice for 15 min. After centrifugation, the supernatants were collected and determined for protein concentration by BCA protein assay kit (Beyotime Biotechnology, China). Equal amounts of proteins were loaded into a 10% or 12% SDS-PAGE for electrophoresis for 1 hour, followed by PVDF membrane transfer (Millipore, Billerica, Massachusetts). The membranes were blocked with 5% non-fat dry milk and then immunoblotted with primary antibodies overnight at 4°C. The PVDF membranes were finally incubated with HRP-conjugated Goat anti-Rabbit IgG antibody (Abcam, Cambridge, MA, ab6721, 1:2000) and followed by detection with electrochemiluminescence (ECL, Millipore).

### Wheat germ agglutinin affinity chromatography

Cell lysate or medium-derived O-glycosylated FGF23 proteins from transfected hFOB1.19 cells were enriched by using wheat germ lectin columns (Vector Laboratories, CA, USA) following manufacturer’s instructions as previously described (14). Samples were finally eluted with elution buffer and concentrated with 3 kDa ultrafiltration tubes (Millipore, MA, USA), and subjected to Western blot analysis.

## Results

### Clinical phenotypes of the patients

**Figure 1:**
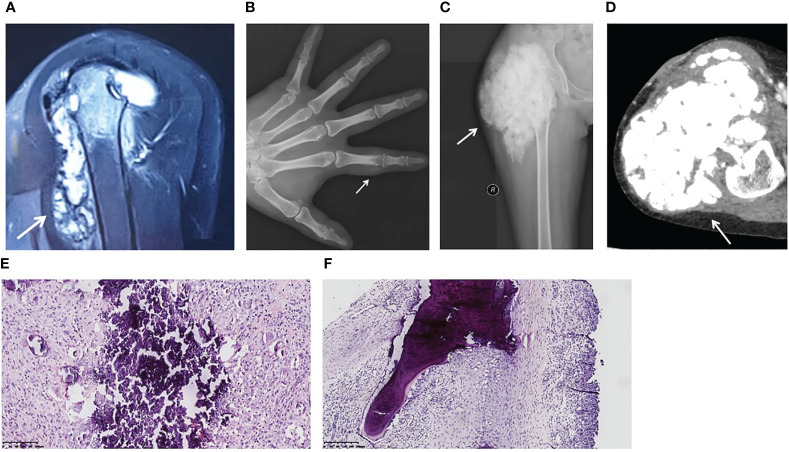
Image findings and pathological features of hyperphosphatemic familial tumoral calcinosis. **(A)** Patient 1. A magnetic resonance image (sagittal T2-weighted fat-saturated) shows a flow-like calcified mass in the left shoulder joint and bone destruction of the left humeral head. **(B)** Patient 2. An anterior-posterior plain X-ray of the left hand reveals a calcified soft tissue mass around the proximal phalanx of the index finger. The proximal interphalangeal joint is intact. **(C, D)** Patient 3. A plain X-ray **(C)** and a computed tomography image **(D)** show a lobulated calcified mass in the right hip and femoral erosion. **(E)** Calcified material is bordered by a proliferation of mononuclear or multinuclear macrophages, osteoclastic-like giant cells, fibroblasts, and chronic inflammatory cells (Patient 3). **(F)** The lesion extends to the bone trabeculae (Patient 1). (H&E, original magnification ×20).

Patient 1 (**Table 2**) was a male aged 30 years. He was referred to our hospital with left shoulder pain and joint motion disorder for 2 years, and was initially misdiagnosed with synovitis. Magnetic resonance images showed an intra-articular lesion with flow-like calcification, and bone erosion was observed in the humeral head (**Figure 1A**). His serum phosphorus level was 2.93 mmol/L (normal range: 0.85-1.51 mmol/L), and his TmP/GFR was 0.96 mmol/L (normal range: 0.80-1.35 mmol/L). He underwent a biopsy, and histopathological examination revealed tumoral calcinosis. Given the complexity of shoulder function, surgical resection was not performed. The patient had been administered sevelamer, a phosphate-binding agent, for 2 months. During follow-up for 24 months, his symptoms did not alter significantly and his serum phosphorus concentration was 2.78 mmol/L. His parents were first cousins. His sister presented with hyperphosphatemia without similar symptoms (**Table 2**), while his parents showed normophosphatemia.

**Table 2:** Clinical features of patients with hyperphosphatemic familial tumoral calcinosis.

Patient	Phenotype	Sex	Age at onset(years)	Duration(years)	Symptoms	Physicalexamination	Mass size(cm)	Phosphorous(mmol/L)	Calcium(mmol/L)	Calcium-phosphorus product	ALP(IU/L)	25-(OH)D(ng/ml)
1	Yes	Male	28	2	Pain, joint motion disorder	Decreased abduction extent of left shoulder	6×2.5	2.93	2.50	90.83	69	24.10
Patient 1’ssister	Suspected	Female	32	/	Asymptomatic	/	/	2.24	2.32	64.44	78	Not detected
2	Yes	Male	24	1	Pain, mass in left index finger, proximal	Swelling, tenderness, decreased range of motion	1.3×1.2×0.5	1.81	2.44	54.76	38	Not detected
3	Yes	Female	4	11	Multiple masses, restricted body growth	Height: 1.40 m, body weight: 30 kg, BMI: 15.3 kg/m^2^. Swelling, warmth, joint movement restriction	18.8×12.2	1.67	2.48	51.36	121	Not detected

ALP, alkaline phosphatase; 25-(OH) D, 25-hydroxyvitamin D; /, not relevant; BMI, body mass index.

Normal range, BMI (15-year-old female), 20.91±3.22 kg/m^2^; serum phosphorous, 0.85-1.51 mmol/L; serum calcium, 2.20-2.55 mmol/L; calcium-phosphorus product, 30-40 mg^2^/dl^2^; ALP, 40-150 U/L; 25-(OH) D, 20.00-40.00 ng/ml.

Patient 2 was a male aged 25 years. He found a painful nodule in his left index finger and experienced movement disorder 1 year before referral. The patient didn’t have early loss of teeth, osteoporosis or fracture. There was no apparent cause or family history. Biochemical tests showed hyperphosphatemia and normocalcemia (**Table 2**). X-rays revealed a calcified soft mass around the proximal phalanx of the index finger (**Figure 1B**).

Patient 3 was a female aged 15 years, without family history. She had undergone three surgeries prior to visiting our hospital. At 4 years of age, she presented with a nodule in her right foot and underwent her first surgery, but the pathological diagnosis from this was not available. Nine days after this surgery, her parents found a hard mass in her right thigh. A second surgery was performed and pathological examination showed calcification. At five years of age, two masses were discovered in her left wrist and right foot, so her third surgery was performed. She was referred to our hospital because of a large firm mass on her right hip and hyperphosphatemia (**Table 2**). No skin lesions or gingivitis were found. X-ray and computed tomography images indicated typical lobulated calcification around the hip and femoral erosion (**Figures 1C, D**).

### Histopathology

The gross specimens for all three patients revealed firm rubbery masses. In Patient 3, the lesion invaded the adjacent joint capsule and bone. On sectioning, the mass consisted of a framework of dense fibrotic tissue containing spaces filled with yellow-gray, pasty, calcareous material or chalky, milky liquid. Microscopically, histopathological features common to the three patients were apparent masses consisting of multiple nodules of calcified material bordered by proliferating mononuclear or multinuclear macrophages, osteoclastic-like giant cells, fibroblasts, and chronic inflammatory cells (**Figure 1E**). In Patient 1, the lesion extended to the bone trabeculae (**Figure 1F**).

### Novel mutation in *FGF23* detected by PCR and sequencing

**Figure 2:**
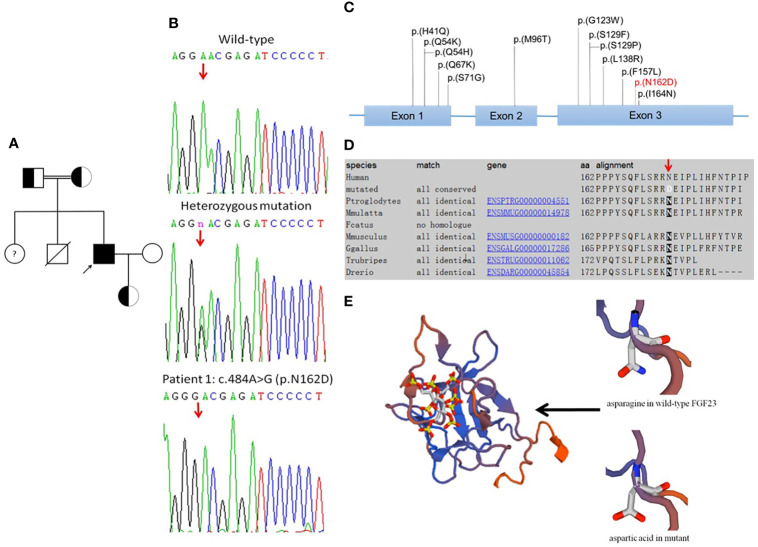
Mutation analysis. **(A)** Pedigree of Patient 1. Squares: male family members; circles: female family members; black symbols: individuals with hyperphosphatemic familial tumoral calcinosis (HFTC). The proband is denoted by the arrow. The patient’s sister is suspected to be affected by HFTC, but her DNA sample was not available. The patient’s brother died at 2 years of age, but had no history related to calcinosis or hyperphosphatemia. **(B)** Sequence of the PCR-amplified exon 3 of the *FGF23* gene. Patient 1 was homozygous for the mutation. The patient’s parents and daughter were heterozygous. The patient’s wife was wild-type homozygous. **(C)** Currently identified *FGF23* mutations associated with HFTC. The mutation identified in the present study is highlighted in red. **(D)** *FGF23* N162 (arrow) is conserved in all available species. The alignment was generated with Mutation Taster. **(E)** Three-dimensional structural representation of the *FGF23* N162D variant. The structure of fibroblast growth factor 23 (FGF23) protein was mutated to N162D in SWISS-MODEL. The arrow shows the position of the asparagine in wild-type FGF23 and the aspartic acid in mutant.

PCR and sequencing were performed in the proband and his family members (unaffected parents, daughter, and wife; **Figure 2A**). In the proband, a novel c.484A>G (p.N162D) homozygous mutation was detected in exon 3 of *FGF23* (**Figure 2B**). His parents and daughter were all heterozygous for the mutation. The unaffected wife was wild-type homozygous. The nucleotide change resulted in an amino acid change at position 162 from asparagine to aspartic acid (p.N162D). This variant was not previously reported, based on searches of the Human Gene Mutation Database and ClinVar (**Figure 2C**, **Table 3**). No nucleotide changes were observed in *GALNT3* or *KL*. In Patient 2 and Patient 3, no genetic variants were identified in the above three genes.

**Table 3:** The mutation sites of *FGF23* in tumoral calcinosis.

Phenotype	Genotype		Possible pathogenic mechanism	PubMed ID
HFTC	c.123C>A	p.H41Q	Decreased secretion of FGF23	19411468
HFTC	c.160C>A	p.Q54K	Decreased secretion of FGF23	18682534
HFTC	c.162G>C	p.Q54H	Not mentioned	33685073
HFTC	c.199C>A	p.Q67K	Decreased secretion of FGF23?	25378588
HFTC	c.211A>G	p.S71G	Decreased secretion of FGF23	15961556
HFTC	c.287T>C	p.M96T	Impaired protein glycosylation?	16151858
HFTC	c.367G>T	p.G123W	Impaired protein glycosylation?	19188744
HFTC	c.386C>T	p.S129F	Decreased binding to FGFR-Klotho complex	16030159
HFTC	c.385T>C	p.S129P	Impaired protein glycosylation?	19837926
HFTC/HHS	c.413T > G	p.L138R	Impaired protein glycosylation	32360901
HHS	c.471C>A	p.F157L	Decreased binding to heparin receptor	24680727
HFTC	c.484A>G	p.N162D	Impaired protein glycosylation	Present study
HFTC/HHS	c.491T > A	p.I164N	Impaired protein glycosylation	32360901

HFTC, hyperphosphatemic familial tumoral calcinosis; HHS, hyperostosis-hyperphosphatemia syndrome.

The *FGF23* N162D mutation was predicted to be damaging and disease-causing by Polyphen-2 and Mutation Taster, respectively, making it a compelling candidate for the disease-causing mutation. N162 was highly conserved among available species (**Figure 2D**). The mutation was predicted by SWISS-MODEL to be highly destabilizing and causative of conformational changes in the three-dimensional protein structure (**Figure 2E**), possibly leading to protein dysfunction.

### Detection of plasma FGF23 concentrations in patient 1 with the N162D mutation

**Figure 3:**
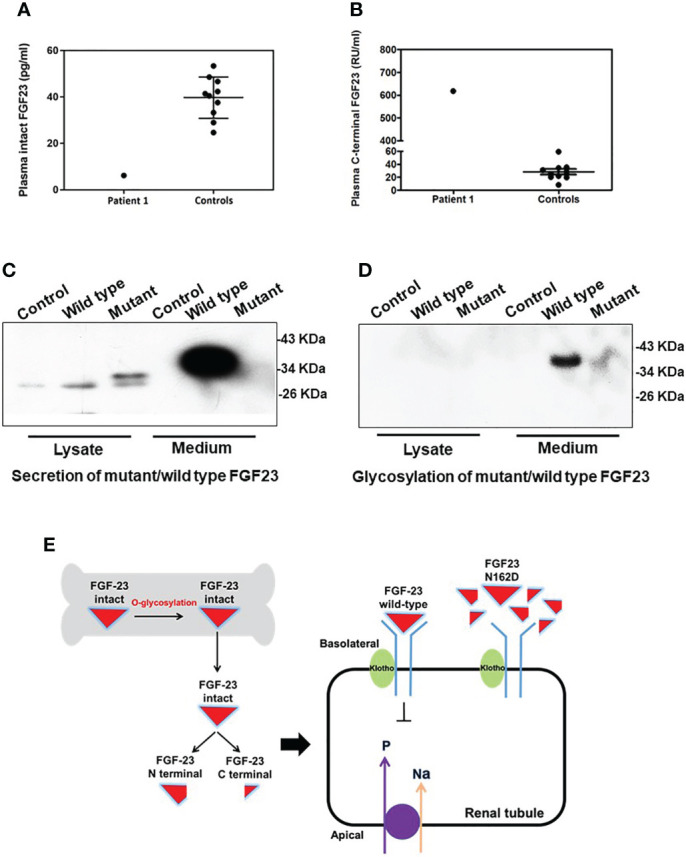
Function prediction and O-glycosylation defect of the *FGF23* N162D variant. **(A, B)** Plasma fibroblast growth factor 23 (FGF23) measurements in Patient 1 showed that the plasma concentration of intact FGF23 **(A)** was decreased, while that of FGF23 C-terminal fragment **(B)** was increased. **(C)** *FGF23* N162D mutation was introduced into osteoblasts hFOB1.19, and proteins in medium and cell lysate were collected respectively. FGF23 protein was detected in both wild-type and N162D mutants in cell lysates using Western Blot. In the medium, however, only wild-type FGF23 protein was detected. **(D)** Wheat germ agglutinin affinity chromatography was used to purify FGF23 glycoprotein. Glycoprotein in medium in wild type, but not in the mutant, was detectable. **(E)** Working model illustrating the probable mechanism of the FGF23 N162D variant. Intact FGF23 binds to FGF receptor 1/α-Klotho complex, thereby inhibiting sodium/phosphate cotransporters NaPi-IIa and NaPi-IIc to decrease phosphate reabsorption. N162D mutation influences FGF23 stability and causes protein proteolysis into N- and C-fragments, resulting in decreased FGF23 binds to receptor complex and increased phosphate reabsorption.

In Patient 1, the concentration of intact FGF23 was 6.2 pg/ml (**Figure 3A**), and markedly decreased compared with the control group (39.8 ± 8.9 pg/ml; range: 24.7-53.4 pg/ml). Meanwhile, the concentration of FGF23 C-terminal fragment was elevated at 617.7 RU/ml (**Figure 3B**), compared with the control group (28.3 ± 13.6 RU/ml; range: 8.2-59.3 RU/ml).

### Detection of FGF23 protein glycosylation and secretion in cells

FGF23 proteins in cell lysates were detected in both wild-type and N162D mutant in hFOB1.19 cells. While the expression levels of WT and mutant FGF23 with small molecular weight (~30 kD) in the lysates of the transfected cells were comparable, the secreted intact FGF23 with molecular weight of 37 kD was only detected in the media derived from the cells expressing WT FGF23 but not in the cells expressing mutant FGF23. (**Figure 3C**). It may be that the level of mutant FGF23 is much lower than that of wild-type FGF23, so the mutant FGF23 band cannot be exposed together with wild-type FGF23 by WB. To determine if the N162D mutation influences O-glycosylation and protein stability and secretion, we performed a WGA affinity chromatography and enriched O-glycosylated fractions from the cell lysates and the culture media. Our analyses revealed that while both wild-type and N162D mutant FGF23 proteins were undetectable in the cell lysates, the glycosylated intact FGF23 protein was only detectable in the media collected from the cell cultures expressing WT FGF23. Our results suggest that N162D mutation might prevent FGF23 from O-glycosylation, impaired protection from protein proteolysis and possible secretion. It should be noticed that an appeared signal in the lane 6, **Figure 3D** was noise (**Figure 3D**).

## Discussion

HFTC is a rare disease characterized by a progressively growing mass of calcified deposits in the periarticular area (1, 15), which is predominantly found in Central Africa and the Middle East (15). In 2000, FGF23 was identified (16) as the most important effector of phosphate balance. FGF23 is produced and O-glycosylated by osteocytes and osteoblasts (**Figure 3B**). Intact FGF23 undergoes posttranslational O-linked glycosylation by N-acetylgalactosaminyltransferase-T3 at a pro-protein convertase cleavage site, protecting FGF23 from cleavage into inactive fragments (17). Thus, in the absence of its O-linked glycosylation, intact FGF23 is readily cleaved and inactivated (1). Intact FGF23 acts *via* FGF receptor 1 along with its co-receptor Klotho to inhibit sodium/phosphate cotransporters (NaPi-2a and NaPi-2c) and 25-hydroxyvitamin D 1α-hydroxylase expression (18). This leads to increased renal phosphate excretion and decreased serum 1,25-dihydroxyvitamin D, thereby lowering the blood phosphate level (19, 20).

Tumoral calcinosis is often misdiagnosed because of its rarity. The differential diagnosis includes bone tumor, tuberculosis, gout, and pseudogout. Although pathological examination is the only means of definite diagnosis, comprehensive consideration of clinical manifestations, biochemical profiles, and imaging features is of great advantage for preoperative diagnosis. The main biochemical manifestations of HFTC include hyperphosphatemia, normal serum calcium concentration, high or inappropriately normal 1,25-dihydroxyvitamin D concentration, and in most cases decreased FGF23 concentration (8, 21). In the present study, the proband presented hyperphosphatemia and increased calcium-phosphorus product. High calcium-phosphorus product enhances soft tissue calcinosis. Evidence has shown an association between calcium-phosphorus product and ectopic calcification (22). In the proband, serum 25-hydroxyvitamin D concentration was normal, serum 1,25-dihydroxyvitamin D level was not assessed because we lacked the required equipment.

The phenotypes of HFTC are heterogeneous. Most subjects have onset of tumoral calcinosis during the first two decades of life (1). However, the patient in our study had late onset in his late twenties. The phenotypes vary widely in severity and involved tissues, ranging from isolated eyelid calcifications to massive periarticular calcifications (23). Ectopic calcinosis is usually located at large joints. The most common locations in descending order are the hip, elbow, and shoulder, with less frequent occurrence in the hand, foot, and spine (7, 24). Our case demonstrated the typical periarticular location and the large size. The sister of the proband did not display overt symptoms, despite characteristic findings on biochemical screening evaluation. In an affected family, variable clinical presentations are often observed among family members (1), suggesting a role of genetic or environmental modifiers, like trauma, in the clinical presentation. Therefore, we recommend long-term follow-up by systematic clinical examinations for potentially affected individuals.

Bone or joint damage caused by ectopic calcification has seldom been described in the relevant literature (1). However, in our study, bone damage occurred at the humeral head, which suggested that bone or joint damage may have been underestimated. Thus, we suggest early detection of bone or joint destruction in tumoral calcinosis to avoid significant symptoms and function loss.

Three pathological phases of tumoral calcinosis were proposed in 1993 (25). In the active phase, the characteristic calcified material caused by hyperphosphatemia is deposited into the soft tissue close to the joints and packaged by a granuloma consisting of macrophages, osteoclastic-like giant cells, fibroblasts, and chronic inflammatory cells as foreign matter. The fibro histiocytic nodules are characterized by fibroblast-like cells and histiocytes devoid of calcification during the early phase (**Figures 1E, F**), and pure calcification nodules without cellular components during the inactive phase are rare. However, we observed these manifestations in all periods for one lesion in the proband.

Our study identified a Chinese family with HFTC which may be associated with a novel *FGF*23 N162D mutation. The proband’s healthy first-degree relatives were heterozygous carriers, consistent with an autosomal recessive mode of inheritance. *FGF23* N162 is highly conserved among species. Online software programs predicted that the *FGF23* N162D mutation may cause conformational changes in the protein. Thus, the mutation was predicted to be pathogenic.

In functional assessment of the *FGF23* N162D mutation, the circulating intact FGF23 level in the proband was low, while FGF23 C-terminal fragment was markedly elevated. These findings indicate that FGF23 function was disrupted by dramatically reduced secretion of intact FGF23 (**Figure 3B**). Wild-type FGF23 is secreted and O-glycosylated by osteocytes/osteoblasts. Intact FGF23 binds to FGF receptor 1/α-Klotho complex, thereby inhibiting sodium/phosphate cotransporters NaPi-IIa and NaPi-IIc to decrease phosphate reabsorption. Previous reports indicated that N162 lies within one of the O-glycosylation sites of FGF23 (26, 27). Previous studies on HFTC showed that several *FGF23* mutations (S129P, Q54K, S71G, L138R and I164N) interfered with the O-glycosylation process of FGF23 (**Table 3**), leading to its degradation and a consequent decrease in intact FGF23 (9, 28), and inefficient glycosylation of FGF23 is the key limiting step in regulating intact biological active FGF23 (29). Consistent with previous studies, we also found a decreased level of intact FGF23 and an increased level of FGF23 C-terminal fragment in the serum of patient 1. Our *in vitro* functional studies uncovered that the N162D mutation resulted in un-glycosylation of FGF23 at the residue of 162 aspartic acid and reduced level of secreted glycosylated form of FGF23, but the cellular intact FGF23 was not changed in the cells expressing mutant FGF23. Our studies provide evidence that N162D mutation influences FGF23 stability and causes protein proteolysis into N- and C-fragments. Whether the mutation also affects protein secretion needs further study in future. Based on our findings, we proposed a mechanism of mutant FGF23 action in the kidneys. N162D mutation might prevent FGF23 from O-glycosylation, which reduced level of secreted glycosylated form of FGF23, influenced FGF23 stability and caused protein proteolysis into N- and C-fragments. Reduced intact FGF23 attenuated the inhibitory effect on sodium/phosphate cotransporters NaPi-IIa and NaPi-IIc, resulting in increased phosphate reabsorption.(**Figure 3E**).

One limitation of this study is that we didn’t find *GALNT3*, *KL* or *FGF23* mutations in Patient 2 and 3. It is possible that the mutation/variants may be in the introns of these three genes or in different genes. Moreover, we performed genetic tests in combination with gene-editing protein function testing to identify whether *FGF23* is associated with HFTC. However, the present study did not perform serum 1,25-dihydroxyvitamin D assays, which may comprehensively clarify the pathogenic role of FGF23 protein in HFTC. These need to be confirmed in further systematic experiment.

In summary, we have presented three HFTC with bone damage. Though rare, patients with tumoral calcinosis may also present with bone damage. Our study identified a novel missense mutation in the *FGF23* gene, and confirmed its damaging role in FGF23 protein O-glycosylation. Our findings expanded the genotype-phenotype spectrum of HFTC, and may providing further understanding of the disease mechanism and assist in the interpretation of the genetic information used for genetic counseling.

## Data availability statement

The original contributions presented in the study are included in the article/Supplementary Materials. Further inquiries can be directed to the corresponding authors.

## Ethics statement

The studies involving human participants were reviewed and approved by Beijing Jishuitan Hospital Institutional Review Board (committee reference number 201803-06). Written informed consent to participate in this study was provided by the participants’ legal guardian/next of kin.

## Author contributions

QZ and WY collected the data and edited the manuscript. BL, HW, DY, ZW, WD were involved in the design of the study and the data acquisition. XC and JY designed the idea and gave the suggestions. All authors contributed to the article and approved the submitted version.

## Funding

This work was supported by grants from the National Natural Science Foundation of China (82070850), Beijing Advanced Innovation Center for Big Data-Based Precision Medicine, Beijing Tongren Hospital, Beihang University & Capital Medical University (BHTR-KFJJ-202014).

## Acknowledgments

We thank all of the subjects for participating in this study.

## Conflict of interest

The authors declare that the research was conducted in the absence of any commercial or financial relationships that could be construed as a potential conflict of interest.

## Publisher’s note

All claims expressed in this article are solely those of the authors and do not necessarily represent those of their affiliated organizations, or those of the publisher, the editors and the reviewers. Any product that may be evaluated in this article, or claim that may be made by its manufacturer, is not guaranteed or endorsed by the publisher.
